# Interleukin 6/Wnt interactions in rheumatoid arthritis: interleukin 6 inhibits Wnt signaling in synovial fibroblasts and osteoblasts

**DOI:** 10.3325/cmj.2016.57.89

**Published:** 2016-04

**Authors:** Khrystyna Malysheva, Karien de Rooij, Clemens W. G. M. Löwik, Dominique L. Baeten, Stefan Rose-John, Rostyslav Stoika, Olexandr Korchynskyi

**Affiliations:** 1 Department of Regulation of Cell Proliferation and Apoptosis, Institute of Cell Biology of the National Academy of Sciences of Ukraine, Lviv, Ukraine; 2 Department of Molecular Biology and Clinical Biochemistry, Institute of Animal Biology of the National Academy of Agrarian Sciences of Ukraine, Lviv, Ukraine; 3 Leiden University Medical Center, Leiden, the Netherlands; 4 Division of Clinical Immunology and Rheumatology, Academic Medical Center/University of Amsterdam, Amsterdam, the Netherlands; 5 Institute of Biochemistry, Christian-Albrechts-University, Kiel, Germany

## Abstract

**Aim:**

To evaluate the impact of previously unrecognized negative interaction between the Wnt and interleukin (IL) 6 signaling pathways in skeletal tissues as a possible major mechanism leading to age- and inflammation-related destruction of bone and joints.

**Methods:**

Luciferase reporter assays were performed to monitor Wnt pathway activation upon IL-6 and tumor necrosis factor-α (TNFα) treatment. Functional contribution of IL-6 and TNFα interaction to inhibition of bone formation was evaluated *in vitro* using small hairpin RNAs (shRNA) in mouse mesenchymal precursor cells (MPC) of C2C12 and KS483 lines induced to differentiate into osteoblasts by bone morphogenetic proteins (BMP).

**Results:**

IL-6 inhibited the activation of Wnt signaling in primary human synoviocytes, and, together with TNFα and Dickkopf-1, inhibited the activation of Wnt response. ShRNA-mediated knockdown of *IL-6* mRNA significantly increased early BMP2/7-induced osteogenesis and rescued it from the negative effect of TNFα in C2C12 cells, as well as intensified bone matrix mineralization in KS483 cells.

**Conclusion:**

IL-6 is an important mediator in the inhibition of osteoblast differentiation by TNFα, and knockdown of *IL-6* partially rescues osteogenesis from the negative control of inflammation. The anti-osteoblastic effects of IL-6 are most likely mediated by its negative interaction with Wnt signaling pathway.

Rheumatoid arthritis (RA) is a chronic systemic autoimmune inflammatory disorder that affects up to 1.8% of adult population of the world. This disease has a significant medical and social impact, since the absence of the effective treatment rapidly leads to reduced quality of patients’ lives and results in disability and even mortality. RA may affect many tissues and organs, but primarily attacks the synovium of joints. The process induces synovitis, synovial hyperplasia with neovascularization, and an excess of synovial fluid causing joint swelling, stiffness, and pain. This leads to a destruction of articular cartilage and multiple erosions into adjacent bones (1). Although RA has been the subject of numerous investigations, the cause of the disease is still unknown and its etiology and pathogenesis remain poorly understood (2).

Multiple cytokines regulate a broad range of inflammatory processes implicated in RA pathogenesis. An imbalance between the pro- and anti-inflammatory cytokine activities favors the induction of autoimmunity, chronic inflammation, and thereby joint damage (3). Tumor necrosis factor-α (TNFα), and interleukins IL-1β and IL-6 play the primary roles in the RA pathogenesis, as well as in other inflammatory diseases (1,4).

IL-6 can promote synovitis and joint destruction by stimulating neutrophil migration, osteoclast maturation, and pannus formation. It may also mediate numerous systemic manifestations of RA, including joint erosions development as a result of IL-6 action toward osteoclasts and osteoblasts differentiation. On the other hand, it plays a positive regulatory role in osteoclast differentiation by inducing the expression of receptor activator of nuclear factor kappa-B ligand (RANKL) on the surface of osteoblasts (5-7).

Several signaling pathways are strongly misregulated in synovial fibroblasts, monocytes, neutrophils, endothelial, and other cells in the joints of RA patients. In particular, recent studies of human rheumatic and orthopedic diseases and specific mouse models with both activating and null mutations of proteins required for the canonical Wnt signaling suggest this signaling cascade as one of crucially important pathways in the regulation of bone formation, maintenance, reparation, and remodeling by regulating osteoblast and osteoclast proliferation and differentiation (8-10). Osteoblast differentiation is predominantly induced by bone morphogenetic proteins (BMP), which are members of the transforming growth factor β (TGFβ) superfamily. However, efficient differentiation of mesenchymal precursors to the osteo- and chondrogenic lineages requires both Wnt and BMP signaling, and the canonical Wnt signaling pathway appears to be master regulator of osteogenesis (11).

Wnt/β-catenin signaling regulates osteogenesis through multiple mechanisms. Wnts repress alternative mesenchymal differentiation pathways, such as adipocyte and chondrocyte differentiation, and promote osteoblast differentiation, proliferation, and mineralization activity while blocking osteoblast apoptosis. β-catenin represses osteoclastogenesis by increasing the ratio of osteoprotegerin (OPG)/RANKL (12). In a healthy skeleton, formation and resorption of adjacent to joints cortical bones are well balanced but inflammatory arthritis leads to an imbalance between these processes. Bone formation is hampered by the TNF-mediated expression of Dickkopf-1 (DKK-1), which suppresses Wnt signals, whereas bone resorption is enhanced by the expression of RANKL – a key factor in the osteoclast differentiation and activation. DKK-1 is critical for joint remodeling. Blockade of DKK-1 relieves Wnt signaling from DKK-1-mediated suppression and induces bone formation mirrored by the growth of osteophytes (13).

In terms of the commitment and differentiation of mesenchymal stem cells (MSC), there is a cooperative crosstalk between the Wnt and BMP pathways (14). BMP signaling is crucial for osteogenesis and skeleton homeostasis during both embryogenesis and postnatal life. The crosstalk between BMP and Wnt signaling is complex in all tissues, and it can be either synergistic or antagonistic depending on the cellular context. In line with the complexity of their crosstalk, BMP and Wnt signaling have opposing effects on the osteoprogenitors, yet for the most part they seem to function cooperatively in osteoblasts and osteocytes.

Although a crucial role for canonical Wnt signaling in skeleton homeostasis has been strongly established, much remains to be discovered in respect to its fine tuning and crosstalk with other pathways in bone (15). The main aim of this study was to evaluate the impact of previously unrecognized negative interaction between the Wnt and IL-6 signaling pathways in skeletal tissues as a possible major mechanism leading to age- and inflammation-related destruction of bone and joints.

## Materials and methods

### Cell culture and ligands

Our studies were performed using mouse NIH-3T3 fibroblasts and mouse mesenchymal precursor cells of C2C12 and KS483 lines. These cells were cultured in Dulbecco's modified Eagle's medium (DMEM, Sigma) containing 10% fetal calf serum (FCS, Sigma). Cells were grown in a 5% CO_2_-containing atmosphere at 37°C. Upon transient transfection, cells were grown in DMEM supplemented with 4% FCS and 16 hours later, they were transferred to fresh DMEM with 10% FCS and addition of appropriate ligands. In particular, cells in appropriate variants were treated with 10 ng/mL of recombinant TNFα, 100 ng/mL of IL-6 in combination with 500 ng/mL of soluble IL-6R. All ligands were purchased from R&D Systems (Minneapolis, MN, USA). Alternatively, in some experiments IL-6/IL-6R combination was substituted with HyperIL-6 fusion protein (10 ng/mL) (16,17).

Primary human synoviocytes (fibroblast-like synoviocytes, FLS) were isolated from synovial biopsies of patients with RA fulfilling the American College of Rheumatology revised criteria for RA (18,19), cultured as previously described (20) and used for experiments between passages 4 and 9, following overnight culture in the medium containing 1% fetal bovine serum (FBS; Invitrogen, Breda, The Netherlands).

### Materials

Short (small) hairpin RNA (shRNA)-expressing constructs are frequently used as a convenient substitution for siRNA specifically targeting gene expression that allows to avoid initial side effects of transfection required for siRNA delivery to the cells. A set of validated shRNA lentiviral constructs that specifically target the expression of mouse versions of *IL-6* mRNAs was purchased as a part of MISSION library from Sigma-Aldrich (St. Louis, MS, USA). Hyper-IL-6, which is a fusion protein of human IL-6 fused to the soluble human IL-6R (16), was prepared as described previously (17).

Plasmids expressing BMP2 and BMP7 full-length cDNA were purchased from Open Biosystems/GE Healthcare (Lafayette, CO, USA). pPGK-mWnt3a-neo plasmid was kindly provided by Drs. Ritsuko and Shinji Takada. MISSION shRNA lentiviral constructs targeting mouse IL-6 were purchased from Sigma-Aldrich (St. Louis, MS, USA). Wnt signaling-specific reporter Bat-Luc and pCMV-DKK-1 plasmids were kindly provided by Dr Stefano Piccolo. pShuttle, pShuttle-CMV, and Easy1 plasmids (components of AdEasy system developed for generation of recombinant adenoviruses described below) were provided by Dr Bert Vogelstein.

### Generation of recombinant adenoviral vectors

BMP2, BMP7, Wnt3a, and Bat-Luc adenoviruses were prepared and grown as described previously (20). Briefly, to generate BMP2, BMP7, and Wnt3a adenoviruses, the full-length cDNA was recloned into pShuttle-CMV plasmid. A pShuttle vector was used in order to make a Bat-Luc reporter adenovirus. Obtained pShuttle or pShuttle-CMV constructs were linearized with PmeI restrictase and recombined with Easy-1 vector. Resulting cosmids were linearized with PacI restrictase and prepared as adenoviruses in HEK-293 cells (20).

### Transient transfection

C2C12 and KS483 cells were split at a density of 1.5 × 10^4^ cells per cm^2^ in 12-well plates. The following day, cells were transiently transfected with plasmid constructs expressing shRNA targeting *IL-6* mRNA or control scrambled shRNA (0.5 µg of total DNA per well). Transfection was carried out using GeneJuice transfection reagent (Merck Millipore, Billerica, MA, USA) following the manufacturer’s protocol. The efficacy of shRNA-mediated knockdown was confirmed by quantitative polymerase chain reaction (PCR) and varied from 6.5 to 8 times for most efficient variants (data not shown).

For luciferase reporter assays, NIH-3T3 cells were split at a density of 1.5 × 10^4^ cells per cm^2^ in 12-well plates. Transfection was carried out using polyethyleneimine reagent (Polysciences, Warrington, PA, USA). pcDNA3 plasmid (Invitrogen) was used as a control vehicle. Co-transfection with pCMV-β-Gal plasmid (Clontech-Takara, Mountain View, CA, USA) was used as an internal control for the efficacy of transient transfection. β-Galactosidase activity in cellular lysates was quantified spectrophotometrcally in 100 mmNa_2_HPO_4_/NaH_2_PO_4_, 1 mm MgCl_2_, 100 mm 2-mercaptoethanol, and 0.67 mg/mL *O-*nitrophenylgalactopyranoside essentially as before (21).

### Luciferase reporter assays

FLS cells were split at a density of 1 × 10^4^ cells per cm^2^ in 12-well plates. The following day, cells were transiently transfected with Bat-Luc reporter adenovirus ([MOI] MO = 200) in a combination with β-Gal adenovirus to be used as an internal control for the efficacy of transduction (MOI = 20). 12 hours later, cellular monolayer was rinsed with a serum-free medium and cells in appropriate variants were transduced with Wnt3a adenovirus or GFP adenovirus at total MOI = 500. After next 12 hours later cellular monolayer was rinsed with a serum-free medium, re-fed with fresh complete culture medium and treated with indicated ligands for next 48 hours. Luciferase reporter assays were conducted using luciferase reporter assay reagent (Promega, Madison, WI, USA) on a Victor 3 machine (Perkin Elmer, Waltham, MA, USA) essentially as before (21).

### Stable infection

C2C12 cells were plated in a complete media overnight. Lentiviral particles were added at MOI = 5 and = 10 in the presence of DEAE-dextran, and cells were incubated for 24 hours. Then, equal amount of fresh media with no lentivirus was added, and the cells were incubated for additional 24 hours. Two days later, transduced cells were selected by adding 3-4 µg/mL of puromycin (Sigma). Obtained puromycin-resistant multi-clonal cultures of C2C12 cells were used. These cells were tested for the presence of the lentiviral p24 using ELISA, and no p24 was detected. An efficacy of shRNA knockdown was confirmed with a Real-Time RT-PCR amplification (data not shown).

### Induction of osteoblast differentiation

C2C12 and KS483 cell lines can be induced to differentiate into osteoblasts by different BMPs, including BMP2 and BMP7. 24 hours after transient transfection, these cells lines were transduced with a combination of adenoviral constructs encoding recombinant BMP2 and BMP7 at the multiplicity of infection (MOI) even to 500 for each one construct (22) to induce a production of BMP2/BMP7 heterodimers along with appropriate homodimers. During osteogenesis assay, C2C12 and KS483 cells were cultured in a differentiation-supporting medium supplemented with 50 µg/mL ascorbic acid for 4 and 10 days, respectively. Starting from day 10 upon induction of osteogenesis, KS483 cells were also supplemented with 5 mM β-glycerophospate for next 8 days totaling in 18 days. Recombinant BMPs were a gift from Dr K. Sampath (Curis Inc., Cambridge, MA, USA).

### Alkaline phosphatase assay

The alkaline phosphatase activity produced by C2C12 was analyzed spectrophotometrically using a π-nitrophenylphosphate (π-NPP) as a substrate (23). Four days after the induction of osteogenesis, the cells were washed twice with 0.4 mL of 1X phosphate-buffered saline (PBS) per well. Afterwards, cells were lysed in 0.2 mL of alkaline phosphatase (ALP) lysis buffer (10 mM glycine, 100 µM MgCl_2_, 10 µM ZnCl_2_, 0.1% Triton X-100) per well and agitated gently for 5 min. Then, 10 µL aliquot of cell lysate was placed into a 96-well plate and ALP activity was revealed with 90 µL/well of ALP assay buffer (100 mM glycine, 1 mM MgCl_2_, 100 µM ZnCl_2_) supplemented with 6 mM π-NPP (Pierce-Thermo Fisher Scientific, Grand Island, NY, USA) (24). Cell lysate samples were mixed gently with a mentioned above reaction buffer and incubated at room temperature until color developed. Optical density was measured at 405 nm (OD_405_) in a 96-well plate reader (BioTek, Winooski, VT, USA).

### Alizarin staining

Histochemical examination of mineral deposition by KS483 cells was performed using conventional staining with Alizarin Red (Sigma-Aldrich) (23). Cellular monolayers were washed with 1X PBS (0.4 mL/well) and fixed in 10% (v/v) formaldehyde at room temperature for 5 min. The monolayers were then washed with deionized H_2_O (dH_2_O) prior to addition of 0.4 mL of 2% Alizarin Red S solution (pH 5.5) per well. The plate was incubated at room temperature for 2-5 min with gentle agitation. After aspiration of the unincorporated dye, the wells were washed twice shortly with 0.4 mL of dH_2_O per well and once with 3 mL of dH_2_O per well while shaking for 5 and 20 min, respectively. Then, the monolayers were stored in 1 mL of 1X PBS and scanned.

### Statistical analysis

Results of luciferase reporter activation and spectrophotometric measurements of alkaline phosphatase activity are expressed as mean ± standard deviation. Data were analyzed using GraphPad Prism 6 program. Statistical differences between experimental variants were assessed by non-parametric two-way ANOVA. Appropriate *P* values were shown in graphs to demonstrate the significance of the results. Only differences with *P*-values lower than 0.05 were regarded as significant.

## Results

### IL-6 together with TNFα inhibits the activation of Wnt pathway in primary human synoviocytes

**Figure 1:**
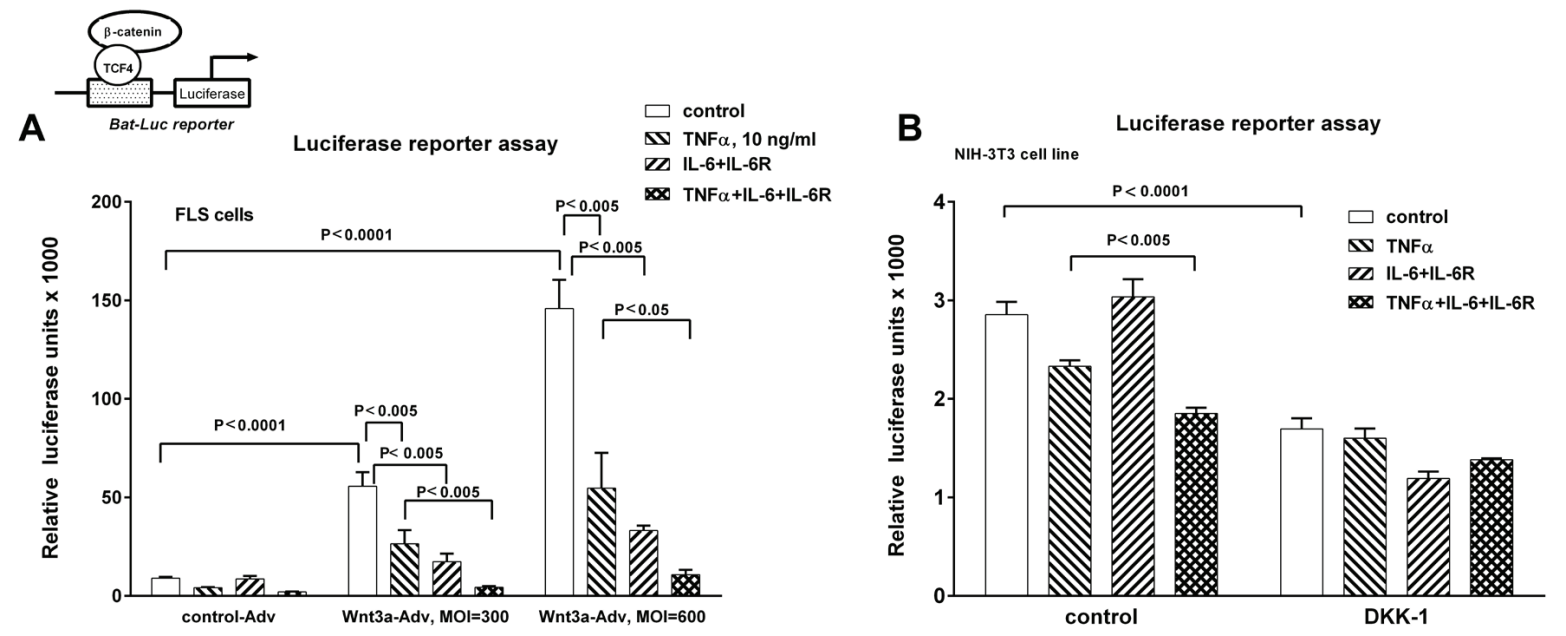
Interleukin-6 (IL-6) together with tumor necrosis factor α inhibits the activation of Wnt signaling pathway in primary synovial fibroblasts (**A**) and IL-6 cooperates with Dickkopf-1 and tumor necrosis factor α (TNFα) in the inhibition of Wnt3a pathway in mouse fibroblasts of NIH-3T3 line (**B**). Wnt response was induced with Wnt3a adenovirus and later cells were treated with a combination of recombinant IL-6 (100 ng/mL) and its receptor IL-6R (500 ng/mL) or 10 ng/mL of recombinant TNFα, or with the combination of all three recombinant proteins for the next 48 hours. Relative luciferase units are shown.

In primary human synoviocytes, both TNFα and *IL-6* inhibited the activation of Wnt signaling induced with the overexpression of Wnt3a adenovirus (Figure 1A). The TNFα inhibition was respectively 2.1 and 2.7 times when Wnt3a adenovirus was used at MOI = 300 and 500. The IL-6 inhibition was 3.2 and 4.4 times more pronounced respectively when Wnt3a was used at MOI = 300 and 500 (Figure 1A). Strikingly, these two proinflammatory cytokines together inhibited the activation of Wnt3a response (respectively 13.1 and 14.5 times for IL-6/IL-6R combination when Wnt3a was used at MOI = 300 and 500, Figure 1A). The experiment was repeated with FLS cells obtained from two more different RA patients with the same trends, but with different magnitudes of induction/inhibition (data not shown).

### IL-6 together with DKK-1 or TNFα inhibits the activation of Wnt pathway in NIH-3T3 fibroblasts

Mouse fibroblasts of NIH-3T3 line were much less responsive to the inhibitory effects of TNFα than human FLS and without stimulation with Wnt3a did not respond to IL-6 (Figure 1B). Nevertheless, in NIH-3T3 cells the combination of IL-6/IL-6R with TNFα showed a cooperative inhibitory effect. We also combined the inhibitory effects of IL-6/IL-6R, TNFα or their combination with the overexpression of DKK-1, a master Wnt signaling inhibitor. IL-6 together with DKK-1 inhibited Wnt response, while a combination of all three agents (IL-6/IL-6R, TNFα, and DKK-1) was less efficient than the combination of IL-6 and DKK-1 (Figure 1B). Moreover, TNFα did not show any effect in combination with DKK-1 or in combination with IL-6/IL-6R and DKK-1 together.

### ShRNA-mediated knockdown of IL-6 expression potentiates and rescues osteoblast differentiation from the negative effect of TNFα

**Figure 2:**
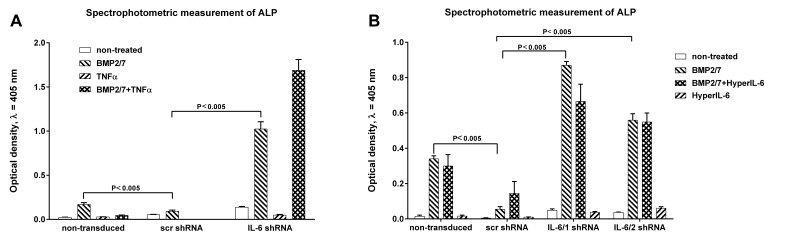
ShRNA-mediated knockdown of *Interleukin 6* expression potentiates and rescues early osteoblast differentiation from the negative effect of tumor necrosis factor α (TNFα) in stable multi-clonal cultures of C2C12 cell line (**A**) and intensifies bone morphogenetic proteins 2/7 (BMP2/7)-induced osteogenesis in stable multi-clonal cultures of KS483 cell line (**B**). Stable lentivirally transduced multi-clonal cultures of C2C12 (**A**) and KS483 (**B**) cells were split into 12-well plates and treated with a mixture of recombinant BMP2 and BMP7 adenoviruses to induce there osteoblast differentiation. 10 ng/mL of recombinant TNFα or 10 ng/mL of recombinant HyperIL-6 were used to modulate osteoblast differentiation. Alkaline phosphatase activity in cell lysates was analyzed spectrophotometrically. Optical density at 405 nm is shown.

While taking into account that RA progression in joints affects also adjacent bones, we further performed an *in vitro* evaluation of functional contribution of IL-6 and TNFα on the inhibition of bone formation using treatment with recombinant cytokines combined with a blocking of *IL-6* expression by shRNA in mouse mesenchymal precursor cells of C2C12. ALP is a widely used marker of early stages in osteoblast differentiation (25-27), and we successfully used it in our study. Treatment of C2C12 cells with TNFα completely inhibited their myoblast differentiation, as well as strongly inhibited BMP-induced osteogenesis (Figure 2A and data not shown).

Transient overexpression of shRNA targeting *IL-6* mRNA, similarly to many other small interfering (siRNA) and shRNA, always induces some off-target interferon response. At the same time, efficient shRNA constructs allowed to partially (anti-*IL-6* shRNA-1) rescue the osteogenic differentiation from negative effect of TNFα. In case of use an anti-*IL6* shRNA-2 TNFα was even converted from an inhibitor into a potentiator of osteogenesis (data not shown and Figure 2A).

Therefore, we generated lentivirally transduced multi-clonal cultures of C2C12 and KS483 cells with stable expression of shRNAs that specifically target the expression of *IL-6* mRNA and scrambled shRNA to be used as a control. shRNA-mediated knockdown of *IL-6* increased BMP2/BMP7-induced osteoblast differentiation (in individual experiments from 2.7 to 6 times compared with the control) in stable multi-clonal cultures of C2C12 (Figure 2A) and KS483 cells (Figure 2B). At the same time, similarly to activation of Wnt pathway in NIH-3T3 cells (Figure 1B), HyperIL-6 treatment alone did not influence early osteoblast differentiation of KS483 cells (Figure 2B). Unfortunately, we were not able to combine BMP2/BMP7 treatment with TNFα due to massive death induced in KS483 cells by TNFα (data not shown). Similar effect was also observed by other investigators with other (pre)osteoblastic cell lines (24,28).

ALP cannot be used as a marker for late stages of osteoblast differentiation for which bone mineral deposition and nodules formation are specific. According to literature and to our preliminary data (not shown), Wnt pathway is activated during late stages of osteoblast differentiation (29). Unfortunately, C2C12 cells cannot undergo late stages of osteoblast differentiation. Therefore, in order to confirm the proper functional outcome of IL-6 inactivation in differentiating osteoblasts, we used KS483 cells that can efficiently follow late stages of osteogenesis (23).

**Figure 3:**
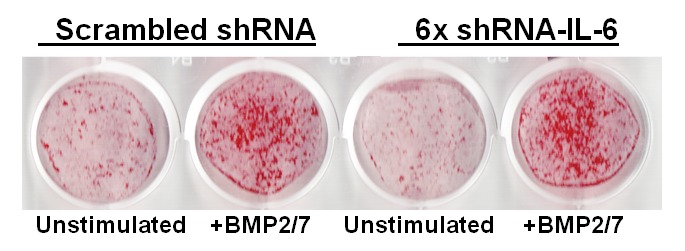
ShRNA-mediated knockdown of *Interleukin 6* expression intensifies bone morphogenetic protein 2/7-induced bone matrix mineralization in KS483 mouse mesenchymal precursor cells. KS483 cells were ectopically transduced with indicated shRNA plasmids (0.5 µg of total DNA per well). Osteoblast differentiation was triggered with the combination of adenoviral constructs encoding recombinant BMP2 and BMP7 for 18 days. Cells were fixed and stained with Alizarin Red. Representative fields (1 × ) are shown.

The treatment of KS483 cells with BMP2/BMP7 strongly intensified their late osteoblast differentiation and overexpression of a combination of 6 versions of shRNA constructs targeting *IL-6* further potentiated osteogenesis. More efficient differentiation was observed through both nodules formation (data not shown) and matrix mineralization when compared with a control scrambled shRNA (Figure 3).

## Discussion

The current study showed for the first time that IL-6 inhibited the activation of Wnt signaling pathway in primary synoviocytes derived from RA patients. The hypothesis about such crosstalk arises from accumulated data taking into account that IL-6 is a well-known target gene for both TNFα and IL-1β (1,4), and Wnt pathway was recently recognized as a master regulator of joint remodeling (13). Even more intriguing is the cooperation between IL-6 and TNFα in the inhibition of Wnt signals because anti-IL-6 therapy appeared to be an important alternative treatment for patients who are not responsive to TNF blockers (30,31).

Interestingly, we found regular NIH-3T3 fibroblasts to be poorly responsive to Wnt3a effects as well as to TNFα treatment. A much lower sensitivity of NIH-3T3 fibroblasts to inhibitory effects of IL-6 combinations with TNFα and with DKK-1 allowed us to speculate that much higher sensitivity of FLS cells represented a cell type-specific feature of these cells that was important for RA development and progression. A similar striking cell type-specific difference is also found in the regulation of presumable RA associated gene, PADI4 (32) and activation of its promoter (data not shown). Moreover, TNFα did not show any effect in combination with DKK-1 and in combination with two other agents (IL-6/IL-6R and DKK-1). Such absence of TNFα effects suggests an overlapping mechanism as it was proposed by Diarra et al (13), who found DKK-1 to be a master regulator of join remodeling. Our speculation about cell-specific feature of FLS cells is also supported with the result of direct HyperIL-6 treatment in KS483 cells, in which we observed a significant potentiation of early osteoblast differentiation upon shRNA knockdown of *IL-6* mRNA. It is still possible that the best results could be obtained with a combined treatment of these cells with HyperIL-6 and DKK-1 since interaction with TNFα-induced DKK-1 could serve as a central mechanism mediating IL-6 effects on the inhibition of osteoblast differentiation. Interestingly, RA progression lowers osteoprotegerin/RANKL ratio and increases circulating RANKL level that correlates positively with C-reactive protein. DKK-1, sclerostin, and osteocalcin levels were also increased with RA progression (30). On the other hand, the anti-IL6 therapy in patients decreases DKK-1 levels in patients’ sera (30). Surprisingly, a similar study performed with the synovial fluids and inflamed joints tissues (31) has found an inverse correlation between the DKK-1 levels and IL-6 levels observed *in vivo* in the inflamed joints. However, these studies only describe the changes without explaining the possible mechanism behind these changes, and our study is the first one that provides a possible explanation for the mechanism.

We showed that knockdown of *IL-6* partially rescued osteogenesis from the negative control of inflammation. Such a result suggests that IL-6 is an important mediator in the inhibition of osteoblast differentiation by TNFα. Despite the fact that IL-6 and TNFα are well recognized as the key cytokines in RA pathogenesis the precise mechanism of how IL-6 and TNFα interaction inhibits bone formation is still not fully understood and a proper understanding of such molecular mechanism(s) for functional contribution of IL-6 and TNFα interaction into inhibition of bone formation is critically important.

Taking into account the known data, it was unexpected that TNFα was converted into an activator of osteoblast differentiation upon shRNA knockdown of *IL-6* expression. This showed that activation/inhibition and regulation of osteogenesis are poorly understood, in particular during inflammation or due to aging. It is a complex system that includes many components and interactions and many of them are still unknown.

**Figure 4:**
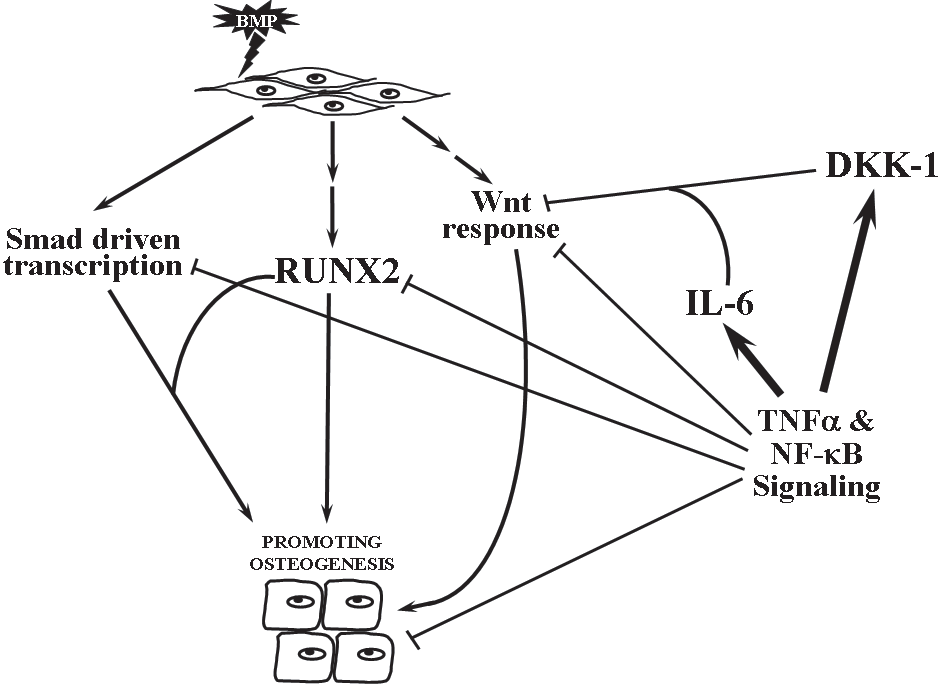
Impact of IL-6/Wnt interaction in joint remodeling. Inflammation inhibits BMP-Smad response and functional activity of its target gene RUNX2, which is important in osteoblast differentiation. TNFα induces both IL-6 and DKK-1 expression. The cooperation of IL-6 and DKK-1 genes is important in inflammatory control of Wnt response and joint remodeling.

We also showed that IL-6 was an important inhibitor of late osteogenesis, which can be explained by the existence of a still unknown direct or indirect negative interaction between IL-6 and Wnt signaling pathways, in which DKK-1 plays an important role (Figure 4) (13,30,31). The Wnt signaling pathway inhibition by this cytokine in skeletal tissues is a possible major mechanism leading to age- and inflammation-related bone and joints destruction.

Thus, our data as well as results of other investigators allow us to hypothesize that the crosstalk between IL-6 and Wnt signaling pathways represents a novel key mechanism for regulation of the homeostasis of joint tissues involved in pathogenesis of RA and osteoporosis progression. Our understanding of the precise molecular mechanisms and functional impact of inhibition of Wnt signaling pathway by IL-6 is crucial for proper understanding of its role in the RA and osteoporosis pathogenesis and progression. Besides this, understanding of these mechanisms can become a basis for development of novel strategies in diagnostics and treatment of this and other related disorders.

In conclusion, IL-6 cooperates with TNFα and DKK-1 in the inhibition of osteogenic Wnt signaling, which is important for joint remodeling. Therefore IL-6 is an important mediator in the inhibition of osteoblast differentiation by the TNFα, and knockdown of *IL-6* partially rescues osteogenesis from negative control of inflammation. The anti-osteoblastic effects of IL-6 in RA are most likely mediated by its negative interaction with Wnt signaling pathway.
